# Is it best to reFOCUS on basic echocardiography in the emergency department?

**DOI:** 10.1186/s13089-018-0102-7

**Published:** 2018-08-15

**Authors:** Pablo Blanco, Gabriela Bello

**Affiliations:** 1 Intensive Care Unit, Clínica Cruz Azul, 2651, 60 St., 7630 Necochea, Argentina; 2 Intensive Care Unit, “Hospital Central de las FF.AA. (DNSFFAA)”, 3060, 8 de Octubre Ave., 11600 Montevideo, Uruguay; 3 “Hospital Policial. (DNAASS)”, 3574 José Batlle y Ordóñez Blvr., 11600 Montevideo, Uruguay

**Fig. 1:**
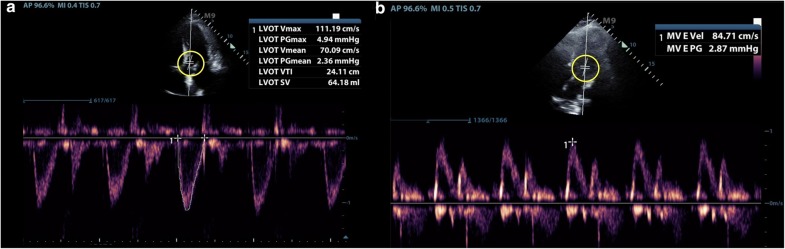
Angle correction (circles) in pulsed-wave Doppler on echocardiography, a practice that is not recommended and was performed in the study of Betcher et al. [1]. **a** Left ventricle outflow-tract flow; **b** transmitral flow. Alignment between the flow and the Doppler must be performed manually and thus requires an excellent technique, without electronic angle correction

Dear Editor,

We read the article of Betcher et al. [1] regarding the feasibility of measuring stroke volume and diastolic function parameters with transthoracic echocardiography (TTE) in the emergency department (ED). While this is clearly stated as a feasibility study, we are honestly questioning the real utility of taking all these measurements, since nearly all patients can be managed without this information on the trenches, reasoning basic echo findings in context with the history and physician examination, without the need of adding complexity, spending extra time, or exposing patients to technique errors or interpretation mistakes. Mentors and trainees must understand (and thus focus on setting the bases of training accordingly) that a well-performed basic echocardiogram (with the addition of the information provided by lung ultrasound, which provides valuable data when showing the mere presence of B lines and/or pleural effusions, answering a simple clinical question in terms of “wet” vs. “dry” lungs [2]) is most of the times enough, and that taking measurements is obviously valid but rarely needed. And when needed, they must be clearly indicated and should be performed by experienced and well-trained practitioners. In this regard, as shown in pulsed-wave Doppler figures, corresponding to Figs. 2 and 3 of the original article (Fig. 1), an angle correction is observed, a practice that is formally not recommended in echocardiography (the angle used should be 0) [3]. This intuitively raises questions about the level of training of the operators in this study as well as of the supervising physicians who consider that exams like these are valid. In addition, all these measurements have inherent limitations, such as LVOT obstruction, a significant aortic regurgitation for LVOT VTI [4], or simply tachycardia for transmitral flow/annulus velocities estimations. In addition, measurement of the diameter of the LVOT to calculate the LVOT area can be readily omitted in practice (avoiding errors derived from the squared nature of the formula), and just using the LVOT VTI to infer the SV and the cardiac output and its variations with treatments [4]. We also honestly have deep doubts regarding whether a 20-min didactic training, practicing in a standardized model or a 1-month point rotation is enough to take these advanced measurements when most real practitioners spend months (or even years) trying to do these accurately. Results of this study are not surprising by considering the aspects expressed above.

In conclusion, in our humble view, we consider that taking measurements may be useless in the ED and that we need to maintain the focus on training and practice moving the needle in favor of basic TTE over advanced TTE. Prioritizing the basic aspects of TTE and lung US is most (if not all) of the times enough in EDs (“visual diagnosis”), and it is better to postpone measurements until they can be performed in a more controlled setting, such as the intensive care unit. Of course, this does not mean that ED practitioners should never perform measurements; of course, they can, but with the requirement of having an excellent level of training, an aspect that requires long months, and even years.
